# Thermal Conductivity of Metal-Coated Tri-Walled Carbon Nanotubes in the Presence of Vacancies-Molecular Dynamics Simulations

**DOI:** 10.3390/nano9060809

**Published:** 2019-05-28

**Authors:** Ravindra Sunil Dhumal, Dinesh Bommidi, Iman Salehinia

**Affiliations:** 1 Department of Mechanical Engineering, Northern Illinois University, DeKalb, IL 60115, USA; rdhumal@niu.edu; 2 Department of Mechanical Engineering, University of Rochester, Rochester, NY 14627, USA; dbommidi@ur.rochester.edu

**Keywords:** metallic coating, multi-walled carbon nanotubes, thermal conductivity, vacancy concentration, phonon and electron heat transfer, phonon density of states

## Abstract

Variation in the thermal conductivity of a metal-coated tri-walled carbon nanotube (3WCNT), in the presence of vacancies, was studied using non-equilibrium molecular dynamics simulations. A Two-Temperature model was used to account for electronic contribution to heat transfer. For 3WCNT with 0.5% and 1% random vacancies, there was 76%, and 86% decrease in the thermal conductivity, respectively. In that order, an overall ~66% and ~140% increase in the thermal conductivity was recorded when 3 nm thick coating of metal (nickel) was deposited around the defective models. We have also explored the effects of tube specific and random vacancies on thermal conductivity of the 3WCNT. The changes in thermal conductivity have also been justified by the changes in vibrational density of states of the 3WCNT and the individual tubes. The results obtained can prove to be useful for countering the detrimental effects of vacancies in carbon nanotubes.

## 1. Introduction

Recent advances in nanotechnology and material design have made it possible for the size of the electronic devices to be reduced to nano/micro level. This often leads to higher power densities, since heat dissipation is restricted to a very small region. Therefore, an efficient thermal management system is central to these small-scale devices. One way to effectively dissipate heat is to employ thermal interface materials (TIMs). Due to their quasi 1-D structure and considerably high thermal conductivity (~600–6000 W/mK) [1,2,3,4,5,6,7,8,9], carbon nanotubes (CNTs) qualify as good candidates for this application. To utilize CNTs as a TIM, the forest of CNTs must be fabricated and located between the heat source and the heat sink. Among various possible configurations, vertically aligned carbon nanotubes (VACNTs), also known as CNT turfs, stand out as the most promising candidate for heat dissipation purposes, due to one dimensional heat transfer.

Phonon scattering, due to inevitable defects that form during CNTs growth, results in significant reduction in their thermal conductivity, and to a larger extent in the overall heat transfer of a CNT turf [10,11,12]. Some of the common defects include 5-7 defect, 5-7-7-5 (Stone Wales), 5-8-5 defect, mono vacancies, di-vacancies, adatoms, etc. [10,13]. Defects in CNTs have been the center of various studies [8,9,10,13,14,15,16,17,18,19]. It has been consistently reported that the effect of vacancy defects on the thermal conductivity of SWCNT is more severe than that of other point defects, such as Stone-Wales (SW) defects and ad-atoms at the same concentration level [9,14,20]. Park et al. [10] and Chen et al. [19] both used reverse NEMD (RNEMD) simulations to report a drop in thermal conductivity of SWCNTs. Park’s study, however, included significant concentration(s) of vacancy defects (up to 2%) and reported over 80% reduction in thermal conductivity as opposed to maximum of three point defects in the study conducted by Chen [19]. A power law decrease in thermal conductivity has also been observed with inclusion of vacancies in CNTs [8,9]. All of the studies probing the effect of vacancies on the thermal conductivity of CNTs, have only been performed on single-walled carbon nanotubes, despite the fact that the majority of CNTs in a CNT turf are multi-walled CNTs (MWCNTs), with 7–10 concentric CNTs [21,22]. Also, as MWCNTs have a greater number of shells, they provide extra pathways for heat transfer [3]. To keep a balance between taking a step closer to reality and performing studies that were not computationally extensive, we have focused on the effect of vacancies on the thermal conductivity of tri-walled carbon nanotubes (3WCNT).

Combining metals and VACNTs has been suggested as a way to improve the thermal properties of the VACNTs as TIMs. The metallization of the CNTs’ ends in VACNTAs has been frequently applied to reduce the interface thermal resistance (ITR) between the VACNTAs and the substrate [23,24]. Despite an order of magnitude improvement on ITR, i.e., approaching 1–2 mm^2^ K/W, metallization of the CNT tips in VACNTAs only affects the ITR between the TIM, the heat source, and the heat sink, hence not alleviating the reduced thermal conductivity, due to vacancies. Filling CNTs with metals has been suggested as another way of improving the thermal conductivity of VACNTAs. Stano et al. [25] outlined the procedure for procuring copper encapsulated VACNTAs and indicated that the resulting composite, by virtue of its superior thermal conductivity, was ideal for thermal management in electronic devices. A molecular dynamics study, conducted by Cui et al. [26], showed above 40% enhancement in the thermal conductivity of gold filled CNTs (nano-cables) over bare CNTs of identical dimensions. An important challenge for filling CNTs with metals is controlling the filling process. Furthermore, as the metal particles only fill in the inner tube, this method only offers a limited design space. To extend the design space, the deposition of metals on the outer tubes of CNTs has been pursued [25,27,28,29,30]. Using electroplating method, Smith et al. [27] and Hua et al. [28] deposited a uniform coating of nickel (Ni) on CNTs. Stano et al. [25] have reported the fabrication of core/shell metal-coated CNT arrays, using oxygen plasma treatment, followed by the infiltration of CNTs with an aqueous supersaturated Cu salt solution. They also claimed that the same procedure can be used to deposit Ni, Fe, Co, and Ag on CNTs in VACNTAs. Electroless deposition [29,31], and chemical vapor deposition [30], have been also reported as possible methods to create a conformal metal coating on a CNT surface. Bommidi et al. [32] have investigated the axial thermal conductivity of a nickel-coated tri-walled CNT using molecular dynamics simulations. The thermal conductivity of the composite material was 50% lower than pristine CNT when 1.2 nm of nickel coating was applied on the CNT. However, the decreasing rate of the thermal conductivity was insignificant for the metal thicker than 1.6 nm. The reduction in the axial thermal conductivity, and by adding metal, was justified by noting that the theoretical thermal conductivity of Ni is much lower than that for CNTs. However, adding a metallic coating on a defective CNT might result in higher axial thermal conductivity as in the presence of vacancies, the thermal conductivity of a CNT might be lower than that of the metal. This study intends to perform such investigation.

While we have only considered the application of metal-coated CNTs as TIMs, metal-coated CNT arrays are also promising in catalysis, energy storage, and sensing applications [33,34,35,36]. For example, while CNTs have been proved as promising materials for sensing applications, carbon may not be the material of choice to be exposed to various environments, resulting in less interest for this material as sensors. Metallic nano-foams show clear advantage in these applications over CNT turfs, however they suffer from microscopically brittle behavior [37]. Metal-coated CNT arrays may resolve the above-mentioned issues with the added benefit of lower density.

Molecular dynamics simulations were performed to study the axial thermal conductivity of tri-walled CNTs in the presence of vacancies. In addition, both phonon and electron contributions to thermal conductivity were considered to investigate the thermal conductivity of metal-coated CNTs when vacancies were present in the CNTs. Due to its ability to form uniform coating on CNTs [27], nickel was chosen for conducting this study. The nickel coating was modeled free of defects, due to the fact that the effect of vacancies on the thermal conductivity of nickel is insignificant, as the heat transfer in metals is mostly controlled by electrons.

## 2. Materials and Methods

Non-equilibrium molecular dynamics (NEMD) simulations were performed in LAMMPS (Large-scale Atomic/Molecular Massively Parallel Simulator) [38] to investigate the thermal conductivity of defective tri-walled CNTs coated with nickel. The results were then compared to those of the models without Ni coating.

The metal’s major contribution to heat transfer would be by virtue of its free electrons. However, NEMD is not capable of considering the electronic heat transfer. Therefore, the two-temperature model (TTM) was implemented in the MD simulations, resulting in accounting for the interactions between phonons and electrons in the system. Wang et al. [39] successfully calculated the thermal conductivity of pure copper within good approximation of the actual value using this technique. Furthermore, they used MD-TTM to calculate the thermal resistance between CNT-Cu interface. NEMD coupled with TTM has also been used for calculating the variation in Thermal conductivity of pristine 3WCNTs with addition of metallic layers around it [32].

For carbon/carbon (C/C) interactions, adaptive intermolecular reactive empirical bond order (AIREBO) potential function was adopted [40]. Nickel/nickel (Ni/Ni) interactions were expressed by the embedded atom method (EAM) potential [41]. Nickel/carbon (Ni/C) interactions were modeled using the Morse type interatomic potential [42]. Detailed description of the interatomic potentials and also the TTM model is available in our recent publication [32].

Thermal conductivity was calculated using the Fourier’s law:

(1) $$k=-\frac{J}{\left(A\cdot \frac{∆T}{∆x}\right)}$$

where *J* is the total heat flux per unit time, *A* is the cross-sectional area, and $\frac{∆T}{∆x}$ is the temperature gradient. To implement this equation, cold and hot heat baths were applied on the two ends of each model and *J* was calculated from Equation (2) [39,43]:

(2) $$J=\frac{Q_{h}-Q_{c}}{2∆t},$$

where the heat current $Q_{h}$, and $Q_{c}$ are the transferred energy from the hot and cold regions, respectively, and ∆t is the time range within which the transferred energies are calculated. The cross-sectional area, *A*, corresponds to $A=\pi \left(R^{2}-r^{2}\right),$ where *R* and *r* are the average Van der Waals radii of the farthest, and the nearest atoms from the CNT axis, respectively [32].

**Figure 1:**
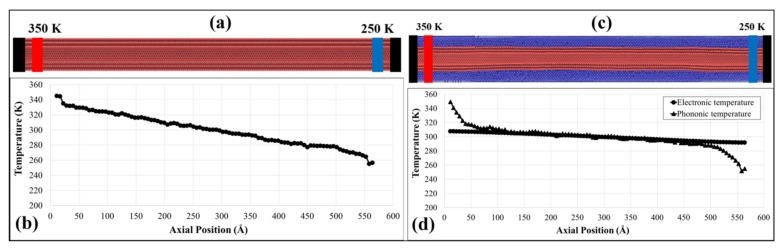
(**a**) Atomic model of a 3WCNT with heat baths and fixed ends, and (**b**) temperature profile along the axis of the 3WCNT due to the heat baths. (**c**) Atomic model of a (1 nm) Ni-coated 3WCNT with heat baths and fixed ends, and (**d**) phononic and electronic temperature profiles along the axis of the model due to the heat baths.

Free boundary conditions were applied to both sides of the models and the structure was divided into 100 grids. Upon energy minimization using conjugate gradient method, the system was equilibrated to 300 K for 175 ps using Canonical (NVT) ensemble. Then, in a Micro-canonical (NVE) ensemble, the third grid, and the third last grid were maintained at 350 K and 250 K, using Langevin thermostat, simulating the thermal heat baths. The choice of the thermostatting method will not have a significant effect on the obtained results, provided that the thermostat regions are kept at their desired temperatures. For the models with the Ni coating, TTM was then enabled and the system was allowed to evolve for 1.25 ns. For using TTM with MD, it was assumed that the temperature of electronic subsystem did not change within a grid. To calculate the temperature gradient, the temperatures of grids between 20 and 80 were linearly fit [32] and averaged over the last 250 ps of each simulation. The grids between 20 and 80 were chosen to ascertain a minimal effect of nonlinearity due to extremely high thermal gradient in NEMD simulations [43]. Values of *J* were calculated every 25 ps (i.e., ∆t in Equation (2)) and were averaged over 250 ps as well. The timestep of 0.5 fs was chosen for each simulation. Figure 1a,c show the schematics of the CNT and the composite, respectively, having fixed ends and heat baths. Figure 1b,d show the respective gradients of temperature along the axis of the models. For the composite model, both electronic and phononic temperatures are plotted in Figure 1d.

The selected tri-walled CNTs for this study were all 57 nm and composed of three coaxial armchair CNTs with chiralities (10,10), (15,15) and (20,20). The armchair CNTs were selected as those have higher thermal conductivity than other types of CNTs, i.e., zig-zag and chiral ones [44,45].

To introduce vacancies in the model, carbon atoms were deleted only from the CNT spanning the middle 20–80% of the its length. This was done to make sure that no atoms were being deleted from the heat baths, thereby not altering the number of thermostat atoms.

Phonon density of states (PDOS) have been used as a common tool for studying the effects of various parameters on the thermal conductivity of CNTs, and the thermal interface resistance between CNTs and other materials [10,46,47,48,49]. We, also used PDOS, in order to explain the observed behavior of the defective and metal-coated CNTs. Phonon density of states refers to the number of phononic modes available as a function of frequency per unit volume [48]. It is obtained by the Fourier transform of the velocity auto-correlation function (VACF), as shown in Equation (3):

(3) $$D\left(f\right)=F\int e^{-2\pi ift}\langle \vec{v}\left(t\right)\cdot \vec{v}\left(0\right)\rangle dt,$$

where, *F* is a normalization factor set equal to 1, *f* is the frequency, and the term in the brackets is the VACF. VACF is obtained by calculating the autocorrelation of the atomic velocities on the consecutive timesteps. Since we are interested in finding axial thermal conductivity, only the velocities in axial direction of the CNT structure were considered for VACF.

## 3. Results and Discussion

**Figure 2:**
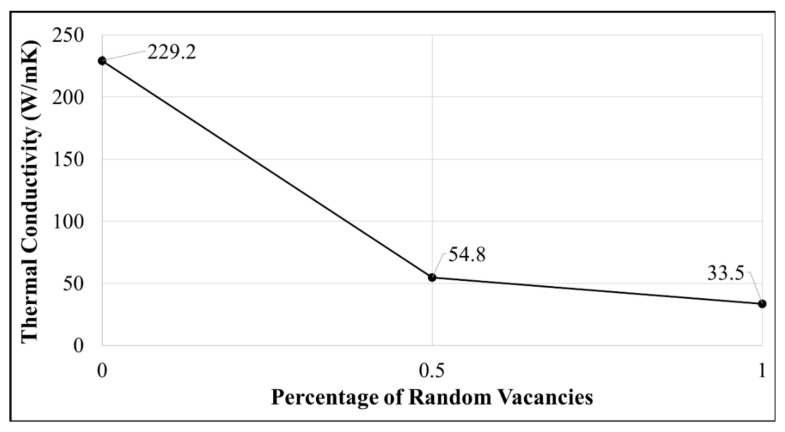
Variation in thermal conductivity of 3WCNTs with various vacancy concentrations. Each data point shows the average of a set of three simulations having three different patterns of (same) vacancy concentration.

Figure 2 shows the variation in thermal conductivity of 3WCNTs with 0.5% and 1% vacancies randomly distributed in the CNTs. To ensure that the distribution of vacancies had no effect on the results, three different distributions were considered for each vacancy concentration. The results show insignificant effect of the vacancy distribution on the thermal conductivities. The thermal conductivity of CNT models with 1% vacancies show almost 86% percent reduction when compared to pristine CNTs. This is in good agreement with the results obtained by Park et al. [10]. For 0.5% vacancies, around 76% drop was observed.

**Figure 3:**
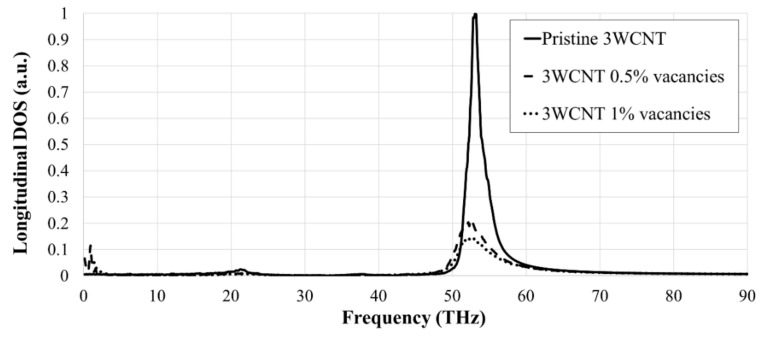
Normalized phonon density of states (PDOS) curves for 3WCNTs with various vacancy concentrations.

Figure 3 shows the variation of the PDOS with frequency for the pristine and defective 3WCNT, indicating the characteristic peak at around 53.1 THz, being in an excellent agreement to the reported value in other literature [26,47,48,50]. There is a significant drop in PDOS peaks, justifying the reduction in the thermal conductivity of the CNTs in the presence of vacancies [51]. The peak’s frequency shows no sign of dependency on the vacancy concentration that is also in alignment with [10].

**Figure 4:**
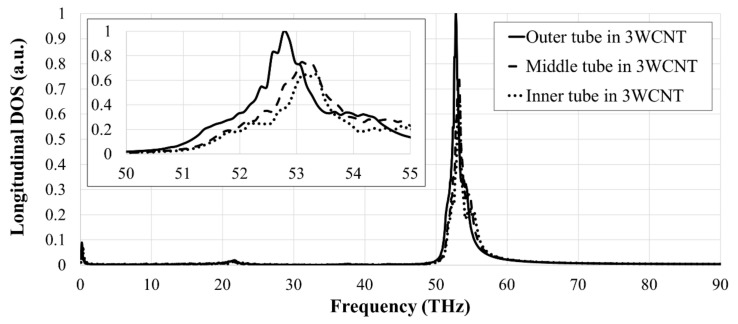
Normalized PDOS curves for each tube in a 3WCNT. The inset shows the curves for the frequency of 50–55 THz.

To better understand the effect of vacancies on the thermal conductivity of 3WCNTs, the heat transfer in each tube in a pristine 3WCNT was evaluated. To perform this study, each nanotube was imposed with a pair of heat baths (hot and cold) of its own. This was done to find out the heat current in each tube separately. These simulations resulted in the lowest heat current for the inner tube, followed by the middle tube, and the highest heat current for the inner tube, i.e., 51.5 eV, 84.85 eV, and 118.1 eV, respectively. The PDOS curves shown in Figure 4 confirm these results as the inner tube possesses the lowest peak and the outer tube has the highest peak. We have found that these individual heat currents are additive, i.e., their summation yields the heat current passing through the 3WCNT as a whole, under the imposition of heat baths at once on all the nanotubes.

**Figure 5:**
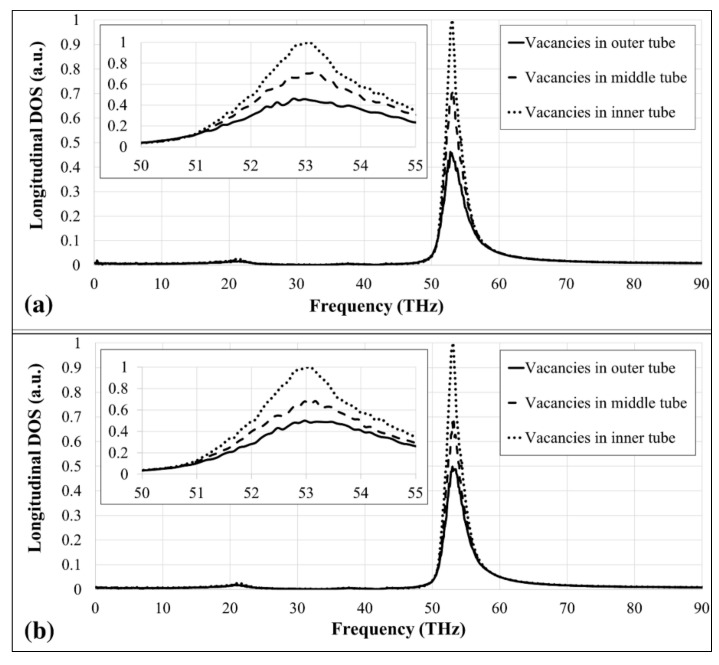
(**a**) Normalized PDOS curves for 3WCNTs with 47 vacancies in each tube (type-I), (**b**) Normalized PDOS curves for 3WCNTs with 0.5% vacancies in each tube (type-II). The insets show the curves for the frequency of 50–55 THz.

The study continues with the inclusion of vacancies in each individual tube to investigate the fundamental effect of the vacancies on the thermal conductivity of 3WCNTs. Two types of simulations were performed; for type-I simulations, the number of vacancies in each individual tube was kept constant, while for type-II simulations, the vacancy concentration in each tube was fixed at 0.5%. Figure 5a shows the variation of the PDOS with the frequency for the 3WCNT when 47 vacancies were positioned in each individual tube. This creates the lowest vacancy concentration in the outer tube, while the vacancy concentration in the entire 3WCNT remains constant. The calculated thermal conductivities for the models with 47 vacancies in inner, middle, and outer tubes are 170.0 W/mK, 154.0 W/mK, and 150.8 W/mK, respectively. PDOS of the 3WCNT when 47 vacancies are in the outer tube shows the lowest peak among other models indicating that this model results in the lowest thermal conductivity. These trends are due to the highest contribution of the outer tube to the heat transfer in a 3WCNT. PDOS for type-II simulations show the same trends as those for type-I’s as illustrated in Figure 5b. The same vacancy concentration in each individual tube resulted in the highest number of vacancies in the outer tube, reducing the thermal conductivity of the 3WCNT the most. The calculated thermal conductivities for the models with 0.5% vacancy concentration in inner, middle, and outer tubes are 170.0 W/mK, 149.0 W/mK, and 125.5 W/mK, respectively. Table 1 lists the values of the thermal conductivity for the considered cases.

As mentioned in the introduction, we intend to check if adding a metallic coating on the outer surface of a defective 3WCNT, can alleviate the deteriorating effect of vacancies on the thermal conductivity of the CNT. To that end, models with metal coatings of various thicknesses on defective 3WCNTs were generated and their thermal conductivities were calculated.

**Figure 6:**
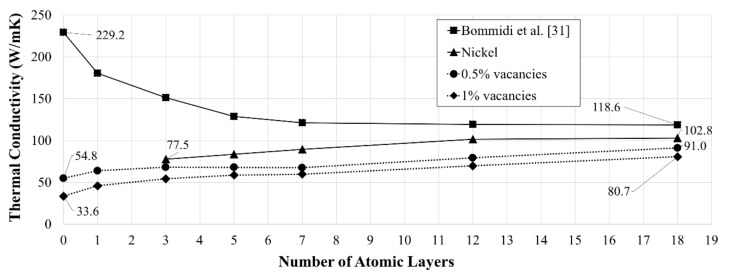
Variation in thermal conductivity of a Ni-coated 3WCNT with varying Ni thickness. Each data point shows the average of a set of three simulations implying three different patterns of (same) vacancy concentration.

**Figure 7:**
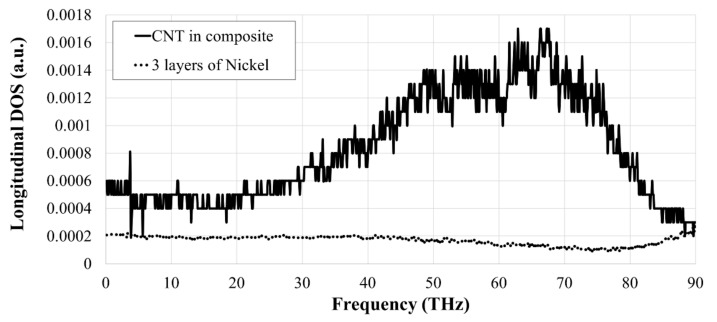
Separate PDOS curves for Carbon and Nickel atoms in the composite with three layers on Nickel.

Figure 6 shows the variation of the thermal conductivity with the number of atomic layers of Nickel coating for various vacancy concentrations, i.e., pristine 3WCNT, 0.5%, and 1%. The curve for the models with a pristine 3WCNT is taken from our recent publication [32]. The thermal conductivity of a bulk nickel is also included in this figure. It is interesting that the thermal conductivity of nickel is size dependent on nano size-scale. This is in good agreement with [52], where the thermal conductivity of Ni nanoparticles was shown to be size-dependent. As a result of the rule of mixture, the thermal conductivity of the metal-coated 3WCNT is between that for 3WCNT, and the thermal conductivity of the nickel tube. A drop in the thermal conductivity, with the coating thickness is linked to the rule of mixture, and also impeding of vibrations of the carbon atoms in the outer tube by the heavier Ni atoms deposited on them, which leads to phonon scattering at the interface of the two elements [32,53]. Figure 7 shows the variation of PDOS for the 3WCNT and also for the nickel coating when 3 atomic layers of nickel was deposited on the CNT. A significant drop is seen for the PDOS of the 3WCNT, when compared to a CNT, without any coating (see Figure 3). Also, there is almost no region of significant shared PDOS for the metal and the 3WCNT to imply phonon-phonon coupling between them [46,49]. Noting that the phonon scattering remains constant with the coating thickness [54], the decrease in the thermal conductivity of the composite with additional nickel is attributed to the intrinsic lower thermal conductivity of the nickel coating [32].

The thermal conductivity of the samples with vacancies increases with the coating thickness. Vacancies in a CNT, with no coating, have resulted in a reduction in the thermal conductivity to a value that is even lesser than the intrinsic thermal conductivity of nickel. Therefore, as per rule of mixture, the addition of nickel helps increase the thermal transport across the structure, thereby, increasing the thermal conductivity of the composite. For 3WCNT models with 0.5% and 1% vacancies, an overall increase of ~66%, and ~140%, respectively, was observed when 3 nm thick nickel coating (18 layers) was deposited around them.

Noting that the models in this work are much shorter than the actual CNTs in a turf of vertically aligned carbon nanotubes, we need to discuss the significance of the length effect on the reported results. It has been repeatedly shown that the thermal conductivity of CNTs increase with length, due to the inclusion of phonons of greater wavelengths [9,10,47,50,55]. However, Park et al. [10] showed that the length dependence of the thermal conductivity vanishes in the presence of vacancies, as no significant change in thermal conductivity was observed even when the length was increased by an order of magnitude, i.e., tenth of micrometer. For such cases, application of metallic coatings can be justified as a means to improve thermal conductivity. Also, the thermal conductivity of the nickel tube is length independent as the mean free path of the thermal transfer in metals is very short. Therefore, the reported results in this work can be applied to CNTs of longer length.

## 4. Conclusions

In this work, we have used non-equilibrium molecular dynamics simulations with two-temperature model (TTM) to study the variation in the thermal conductivity of a metal-coated tri-walled CNT (3WCNT), for different vacancy concentrations. TTM has been specifically employed to capture the electronic contributions to heat transfer in the metallic coatings. 

We have found that the outermost tube in the 3WCNT, by virtue of the highest heat current carrying capacity, contributes the most towards heat transfer. The effect of tube specific vacancies on the thermal conductivity of 3WCNT was explored, by imposing the same number of vacancies (type I), and the same vacancy concentration (type II) on each tube. For both cases, the thermal conductivity of the 3WCNT was found to be the most sensitive to the vacancies in the outermost tube. On the other hand, the thermal conductivity of 3WCNT was affected the least when vacancies were only distributed in the innermost tube. To better understand the variation in thermal conductivity, we have also looked into changes in phonon density of states with the frequency of individual tubes and for the entire 3WCNT.

For the standalone 3WCNT model, the introduction of 0.5% and 1% random vacancies (throughout the three tubes) depreciated the thermal conductivity by over 76%, and 86%, respectively. The thermal conductivities of the defective CNT were lower than the thermal conductivity for the metal coating and thus, the addition of metallic coatings steadily increased the thermal conductivity of the metal-CNT composite. There was an overall ~66% and ~140% increase in the thermal conductivity, with the addition of 3 nm (18 layers) thick metallic coating for 3WCNT, with 0.5%, and 1% vacancies, respectively. Since, the intrinsic thermal conductivities of the metal coating and the defective CNT are understood not to change with length, the obtained results can also be applied to longer CNTs.

## Figures and Tables

**Table 1:** Values of thermal conductivity in unit of W/mK for 3WCNTs when vacancies are only positioned in one individual tube. The numbers in the parentheses are the total number of vacancies.

Type-I (Same Number of Vacancies on Each Tube)			Type-II (0.5% Vacancy Concentration on Each Tube)		
Inner (47)	Middle (47)	Outer (47)	Inner (47)	Middle (72)	Outer (94)
170.4335	153.993	150.8788	170.4335	148.9752	125.5104
